# DNA methylation levels are highly correlated between pooled samples and averaged values when analysed using the Infinium HumanMethylation450 BeadChip array

**DOI:** 10.1186/s13148-015-0097-x

**Published:** 2015-07-31

**Authors:** Cristina Gallego-Fabrega, Caty Carrera, Elena Muiño, Joan Montaner, Jurek Krupinski, Israel Fernandez-Cadenas, On behalf of Spanish Stroke Genetics Consortium

**Affiliations:** Stroke pharmacogenomics and genetics, Fundació Docència i Recerca Mutua Terrassa, Hospital Universitari Mútua de Terrassa, C/ Sant Antoni 19, 08221 Terrassa, Barcelona Spain; School of Medicine, University of Barcelona, Barcelona, Spain; Neurovascular Research Laboratory, Institut de Recerca, Universitat Autònoma de Barcelona, Hospital Vall d’Hebron, Barcelona, Spain; Servicio de Neurología, Hospital Universitari Mútua Terrassa, Terrasa, Barcelona Spain; School of Healthcare Science, Manchester Metropolitan University, Manchester, UK

## Abstract

**Background:**

DNA methylation is a heritable and stable epigenetic mark implicated in complex human traits. Epigenome-wide association studies (EWAS) using array-based technology are becoming widely used to identify differentially methylated sites associated with complex diseases. EWAS studies require large sample sizes to detect small effects, which increases project costs. In the present study we propose to pool DNA samples in methylation array studies as an affordable and accurate alternative to individual samples studies, in order to reduce economic costs or when low amounts of DNA are available. For this study, 20 individual DNA samples and 4 pooled DNA samples were analysed using the Illumina Infinium HumanMethylation450 BeadChip array to evaluate the efficiency of the pooling approach in EWAS studies. Statistical power calculations were also performed to discover the minimum sample size needed for the pooling strategy in EWAS.

**Results:**

A total of 485,577 CpG sites across the whole genome were assessed. Comparison of methylation levels of all CpG sites between individual samples and their related pooled samples revealed highly significant correlations (rho > 0.99, *p*-val < 10^−16^). These results remained similar when assessing the 101 most differentially methylated CpG sites (rho > 0.98, *p*-val < 10^−16^). Also, it was calculated that *n* = 43 is the minimum sample size required to achieve a 95 % statistical power and a 10^−06^ significance level in EWAS, when using a DNA pool strategy.

**Conclusions:**

DNA pooling strategies seems to accurately provide estimations of averaged DNA methylation state using array based EWAS studies. This type of approach can be applied to the assessment of disease phenotypes, reducing the amount of DNA required and the cost of large-scale epigenetic analyses.

## Background

Epigenetics refers to the stable, heritable and reversible modifications in DNA expression associated with transcriptional regulation without alterations in the nucleotide sequence [1]. Epigenetic processes such as DNA methylation (DNAm), histone acetylation/deacetylation, non-coding mRNA expression and chromatin conformational changes [2] are essential for normal cellular development and differentiation. They have also been linked to some monogenic and complex human diseases [3, 4]. Nowadays DNA methylation is one of the most studied epigenetic modifications [5, 6] and alterations in methylation have been linked with some disease processes such as different types of cancer [4, 7, 8], as well as with aging and exposure to tobacco smoke [9–12].

Some of the most important technologies used to detect DNA methylation are: deep sequencing, high-throughput deep sequencing and array-based genome-wide studies such as Epigenome Wide Association (EWAS) [13].

In the “omics” era, Genome-wide Association Studies (GWAS) have been widely used to discover the genetic polymorphisms associated with human diseases. These studies have been more successful in finding genes associated with complex diseases, compared to classical candidate genes studies. However, GWAS needs higher sample sizes and specific arrays that increase project costs. Several papers have observed that the use of pooling strategies decreases the cost of GWAS, while providing similar results to individual sample analysis [14].

Pearson and colleagues reported that pooling-based GWAS was theoretically effective in identifying genetic associations in different types of disease [15]. Applying these methods to experimental case–control data, they also demonstrated the successful identification of previously published susceptible loci for a rare monogenic disease, a rare complex disease and a common complex disease. In addition, Gaj et al. confirmed previously reported loci for colorectal cancer and prostate cancer in a Polish population, with a pooled-based strategy using GWAS [16].

Epigenome-wide association studies (EWAS) use the same strategy as GWAS, but for epigenetics. EWAS use array-based genotyping technology to detect the methylation levels at CpG sites across the genome. EWAS of human diseases are becoming increasingly common [4, 7, 17, 18]. Like GWAS, the EWAS are hypothesis-free approaches to finding differentially methylated sites instead of different allele frequencies. Nevertheless, pooled DNA strategies might be an affordable alternative that reduces study costs in array-based EWAS.

No current studies have analysed the accuracy of DNA pooling strategies in array-based EWAS. Our aim is thus to analyse the pooling strategies in EWAS studies in order to determine the effectiveness of these approaches in studying DNA methylation patterns in human samples.

In the present study, data from 20 individual DNA samples and 4 pooled DNA samples, analysed with the Illumina Infinium HumanMethylation450 BeadChip, were used to estimate the feasibility of the pooling approach, comparing the results of the individual samples to the results of the DNA pools of the same samples.

## Results and discussion

### Quality control

**Fig. 1:**
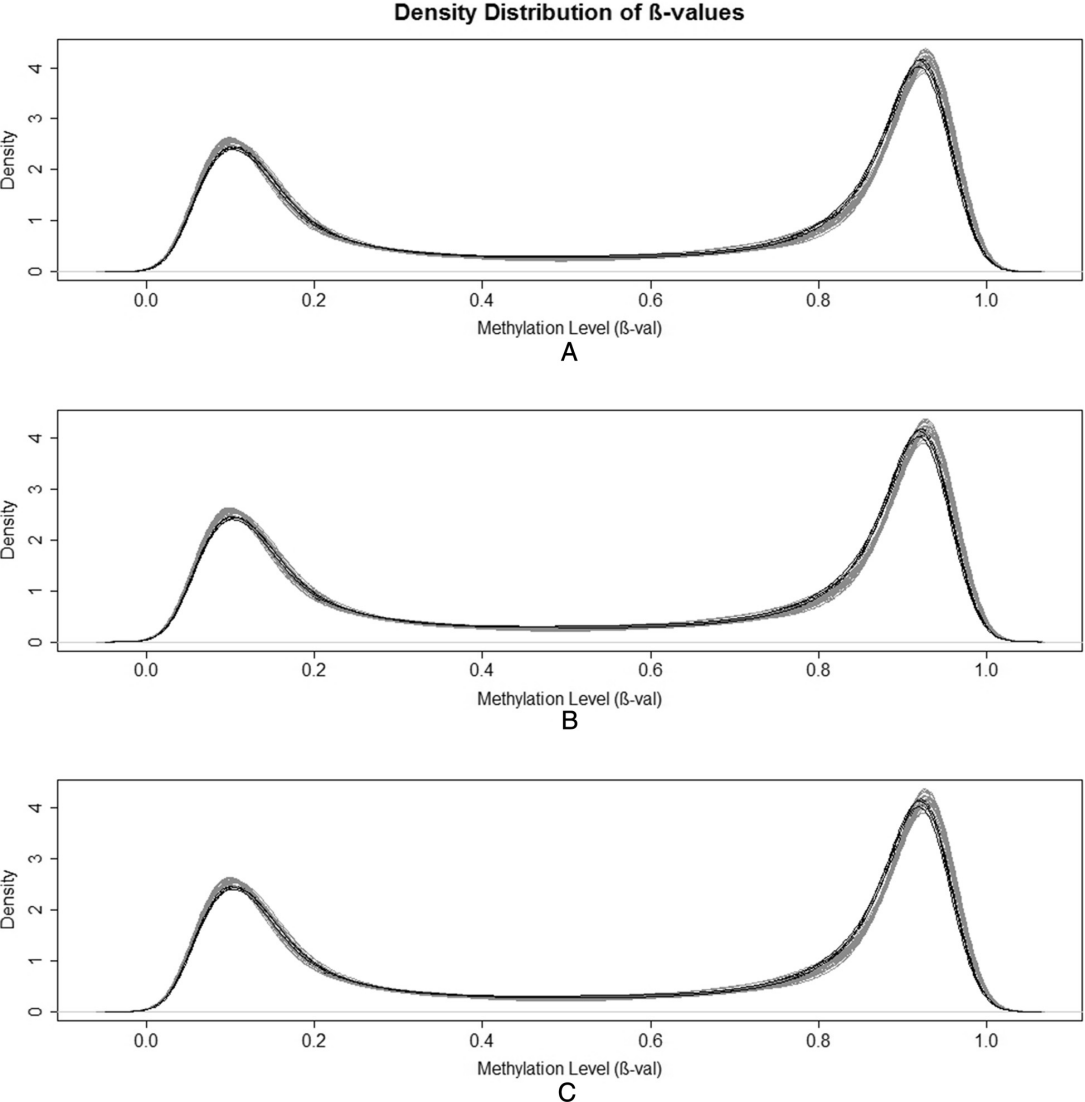
Density distribution of β-values. **a** Density distribution of β-values across all the 485,577 CpG sites of the 20 samples and the four pools. **b** Distribution of five samples of group A and their respective pool. **c** Distribution of five samples of group B and their respective pool. The black lines represent the distribution of pools and the grey lines represent the distribution of samples. The X-axis represents the average methylation β-value and the Y-axis the density

A total of 485,577 CpG sites across the whole genome were assessed using the Illumnia HumanMethylation450 BeadChip, in 20 individual samples and 4 DNA pools. First, the distribution of methylation level was evaluated for all samples, with DNA pools showing the same behaviour as individual samples (Fig. 1). Before the quantile normalization, 33,301 CpG sites and no samples were removed due to QC issues.

### Correlations

**Fig. 2:**
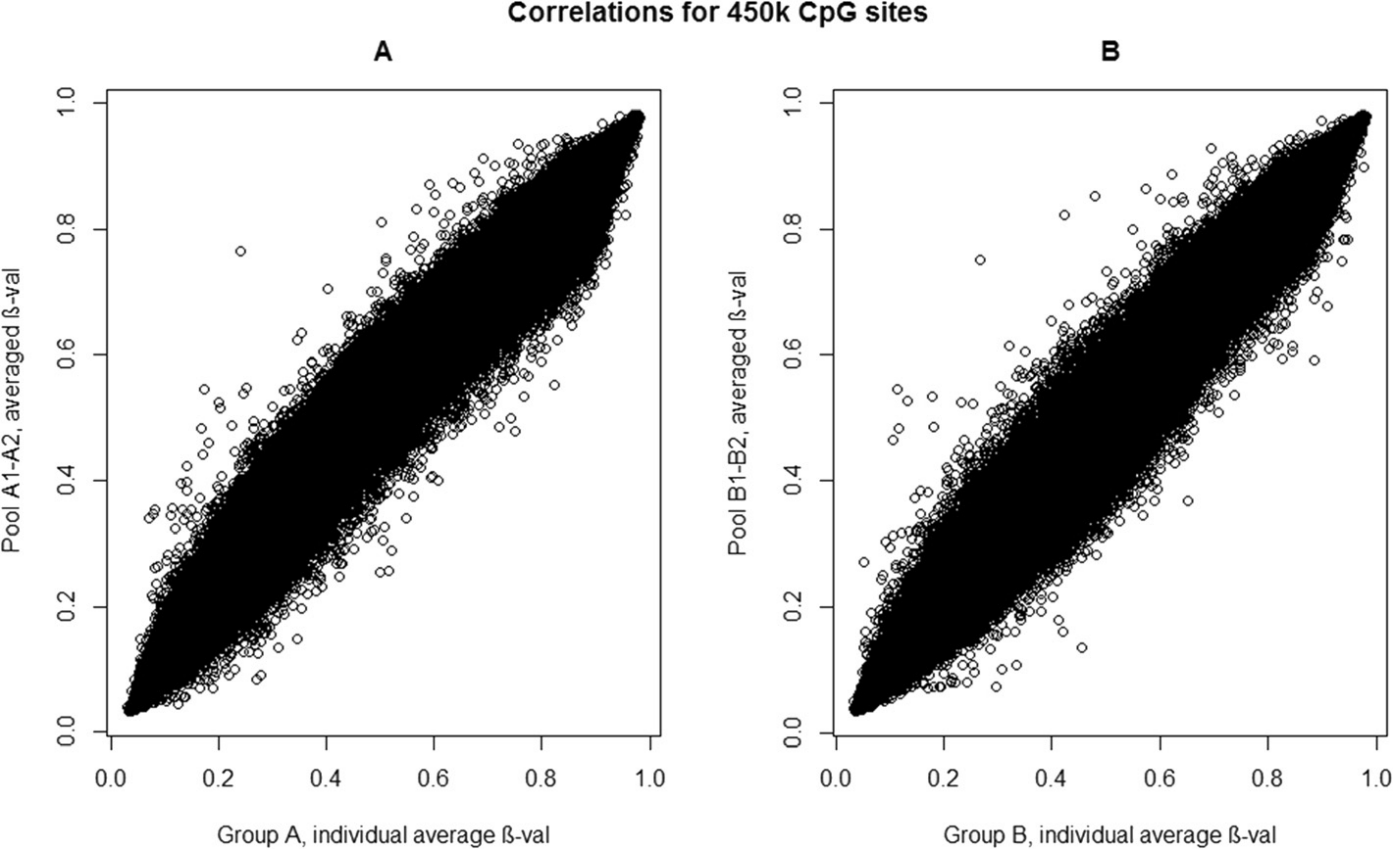
Correlations for 450 k CpG sites. Correlations between averaged β-values of individual samples and averaged β-values of pools. **a** Correlations for group A samples and their pools. **b** Correlation for group B samples and their pools. The X-axis represents the average methylation β-value for individual samples; the Y-axis represents the average methylation β-value for pools

Data revealed highly significant correlations (*p*-value < 10^−16^, Spearman’s test) after comparing the data generated from the pooled DNA samples with the averaged results of the individual samples. Group A and group B samples were studied separately with their respective pools, the obtained correlations were rho = 0.9922 (*p*-values < 10^−16^) for group B and rho = 0.9914 (*p*-value < 10^−16^) for group A. (Fig. 2).

**Fig. 3:**
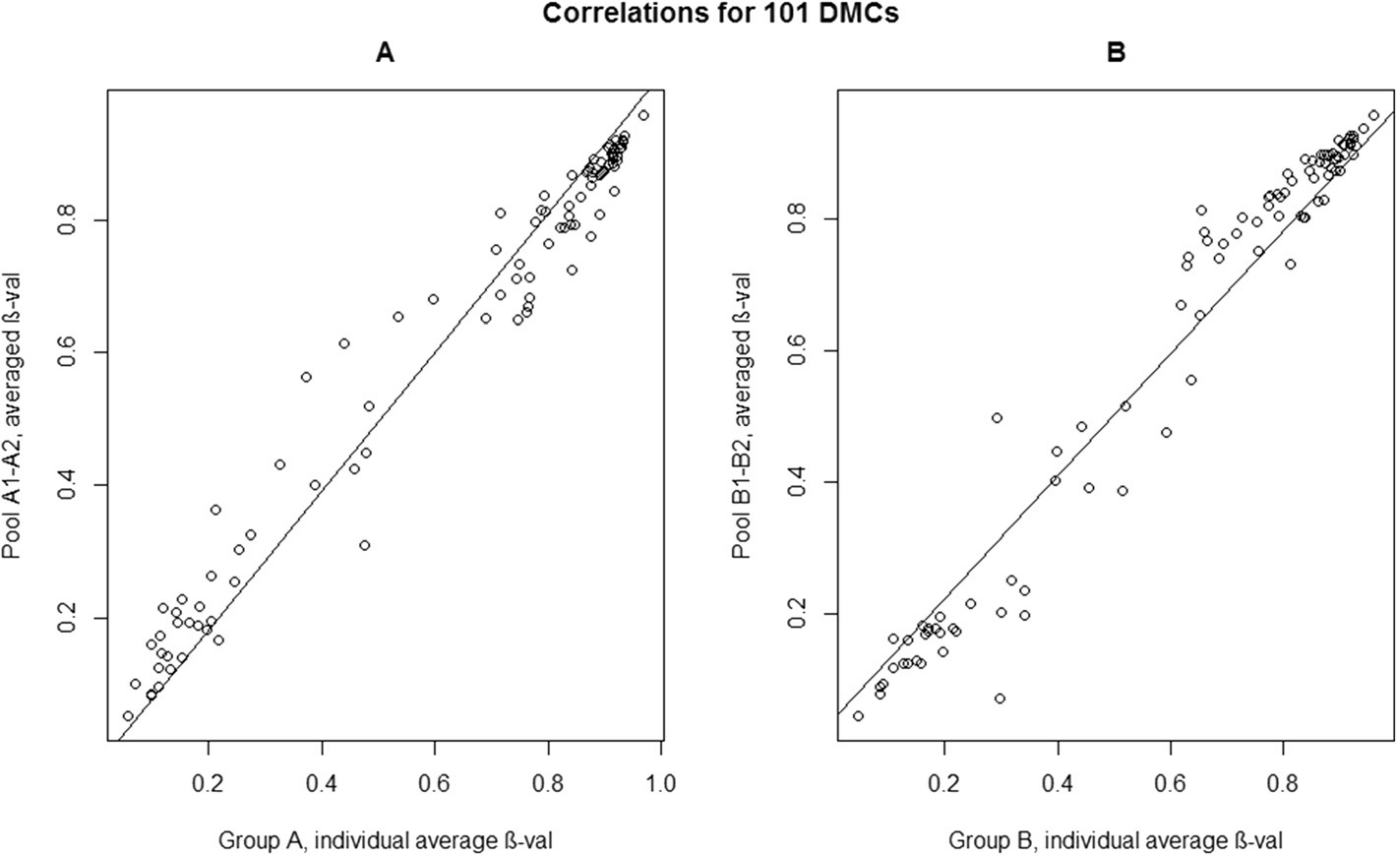
Correlations for 101 DMCs. Correlations between averaged β-values of individual samples and averaged β-values of Pools, for the 101 most significant DMCs. **a** Correlations of 101 CpGs for group A samples and their pools. **b** Correlations of 101 CpGs for group B samples and their pools. The X-axis represents the average methylation β-value for individual samples; the Y-axis represents the average methylation β-value for pools

In addition, a second confirmation test was performed to assess the potential to estimate accurate β-values in the most significant differentially methylated CpGs when using a DNA pool strategy in EWAS. A comparison between all CpGs between group A and group B was performed and the most significant CpG sites (*n* = 101), *p*-val < 10^−05^, were selected. Highly significant correlations (*p*-val < 10^−16^) were also observed when analysing the 101 selected CpGs between group A and their pooled samples and between group B and their pooled samples (Group A: rho = 0.9808, *p*-value < 10^−16^; Group B: rho = 0.9872, *p*-value < 10^−16^) (Fig. 3).

### Sample size

Using values from the most significant DMC of pooled samples in the EWAS study, the optimum sample size to reach a 95 % statistical power and a 10^−6^ significance level, should be from 43 to 100 pooled samples per condition, considering Cohen’s d effect sizes of 1.5 to 0.95 respectively.

The accuracy and reproducibility of DNA pools for methylation array, using the Illumina Infinium HumanMethylation450 BeadChip array, was investigated by comparing data obtained from individual samples and the same samples after they had been pooled.

Our data indicate that the DNA methylation profile (β-values of CpG sites) from the pooled DNA samples using array technology are highly consistent with those obtained from the individual samples, even when evaluating the most significant DMCs separately (Group A: rho = 0.9808, *p*-value < 10^−16^; Group B: rho = 0.9872, *p*-value < 10^−16^).

A previous study analysing pooling strategies in methylation studies demonstrated that pools could be an alternative technique when small amounts of DNA are available or when a reduction in cost is necessary to undertake the experiments. In the study, Docherty et al. showed a correlation between 89 individual samples and 4 pool samples in 205 CpG sites spanning 9 genomic regions using Sequenom EpiTYPER [19]. The overall correlation value in the study was 0.95 with a p-value < 2.210^−16^, similar to the results that we observed. However, in our study we found that pooling strategies can be also performed assessing whole genomes in array-based EWAS experiments, analysing more than 450,000 CpG sites. This finding expands the possibilities of Genome Wide studies in epigenetics. In a pooling-based GWAS study, Pearson et al. demonstrated successful identification of published genetic susceptibility loci for some human diseases: *APOE-ε4* in Alzheimer disease, *MAPT* in progressive supranuclear palsy and *TSPYL* in sudden infant death with dysgenesis of the testes syndrome (SIDDT) [15]. In EWAS we have yet to confirm whether previously reported genes can be found using pooling strategies. However, the higher correlation of the methylation levels between pools and individual samples indicates that the pooling strategies in EWAS are an accurate and interesting strategy to reduce time costs and DNA amount in such experiments.

Even though a DNA pooling strategy has important advantages, there are several drawbacks that have to be considered in the study design. Pool construction has to be really precise. DNA quantities have to be really accurate to assure that each sample in the pool provides equal quantities of DNA in order to minimize technical errors that may alter the estimated methylation levels [14, 20]. Only mean methylation levels, and not individual methylation data, can be obtained from pooled samples. In addition, adjusting for covariates is almost impossible, unless pooled samples are very homogeneous. Population stratification needs to be excluded. Furthermore, the error rate tends to be higher in pooled samples compared to individual ones [21]. It is also important in the study design for EWAS with pooled samples to take into account the sample size needed to compute DMCs with confidence. According to the results obtained in our study, we suggest analysis of at least *n* = 43 pooled samples per condition in order to achieve a 10^−06^ significance level and 95 % statistical power, considering a Cohen’s d effect size =1.5. However, this number may vary depending on a study’s characteristics.

In summary, this is the first study that analyses a pooling strategy in EWAS approaches, it found that this strategy is an acceptable alternative to regular individual EWAS analysis, mainly in specific situations such as when lower quantities of DNA are available, or in studies with a limited budget.

## Conclusions

The analysis of the data generated by 450,000 CpG sites across the whole genome in 20 individual samples demonstrates that DNA pooling strategies can be used to provide estimations of averaged DNA methylation state using the Illumina Infinium HumanMethylation450 BeadChip array. This approach may be useful to highlight genome regions to be studied in further epigenetic analysis, reducing the costs and the amount of DNA required.

## Methods

### Sample selection and pool construction

**Fig. 4:**
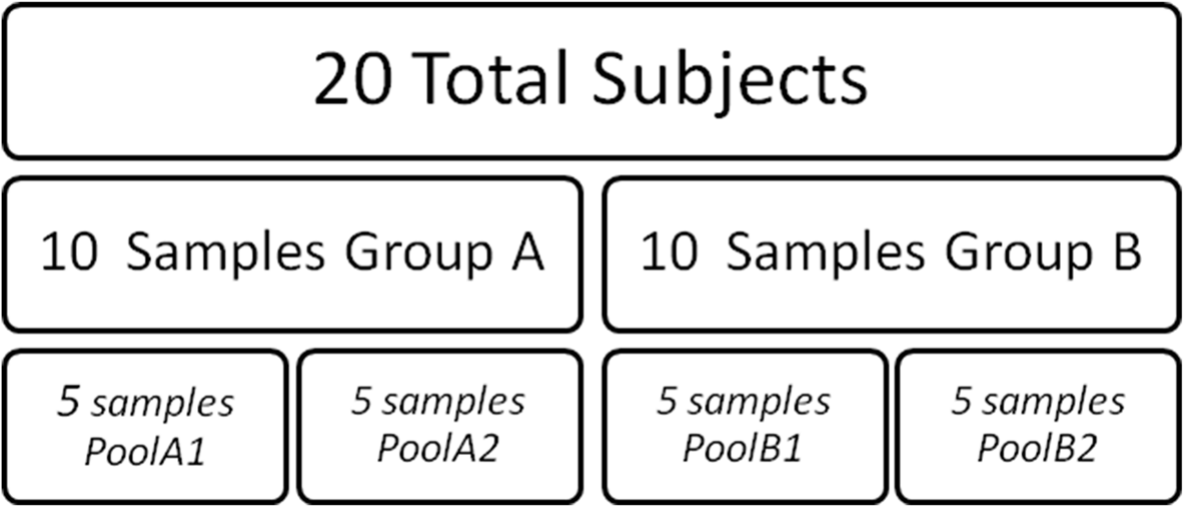
Study design. A total of 20 subjects from the biobank study were selected for this study, 10 of them form group A and 10 form group B. Two pools were created from group A, with 5 samples in each one (PoolA1 and PoolA2), and two more pools of 5 samples were created from group B (PoolB1 and PoolB2)

A total of 20 subjects from our biobank were selected. Of these 20 subjects, 10 were ischemic stroke patients with vascular recurrence (this selection was performed randomly from the patients with vascular recurrence) (group A). These patients were then matched one-to-one with 10 ischemic stroke patients without vascular recurrence (group B). The matching categories were age (±7 years), sex, TOAST classification [19] and recruitment hospital. Next, two pooled samples were constructed with samples from group A (PoolA1 and PoolA2), and two pooled samples were constructed with samples from group B (PoolB1 and PoolB2), as described in Fig. 4. PoolB1 included the matched samples of PoolA1 and correspondingly PoolB2 included the matched samples of PoolA2. All individuals were Caucasian, while 16 were males and 4 were females, mean age was 71 ± 8 years (Table 1).

**Table 1:** Population characteristics

		Total	Group A	Group B
Gender	*N*	20	10(50 %)	10 (50 %)
	Age	72,25 ± 8.4	72,5 ± 8.4	72 ± 8.7
	Male	16 (80 %)	8 (40 %)	8 (40 %)
	Female	4 (20 %)	2 (10 %)	2 (10 %)
TOAST^a^	Atherothrombotic	8 (40 %)	4 (20 %)	4 (20 %)
	Undetermined	4 (20 %)	2 (10 %)	2 (10 %)
	Unknown	2 (10 %)	1 (5 %)	1 (5 %)
	Lacunar	6 (30 %)	3 (15 %)	3 (15 %)

Demographic and clinical variables of the studied population

^a^TOAST classification of ischemic stroke

### DNA purification and sample pooling

Total genomic DNA was extracted from whole blood samples using the Gentra Puregene Blood Kit (Quiagen, Hilden, Germany) following the manufacturer’s instructions. The samples were maintained at −20 °C until the EWAS analysis.

**Fig. 5:**
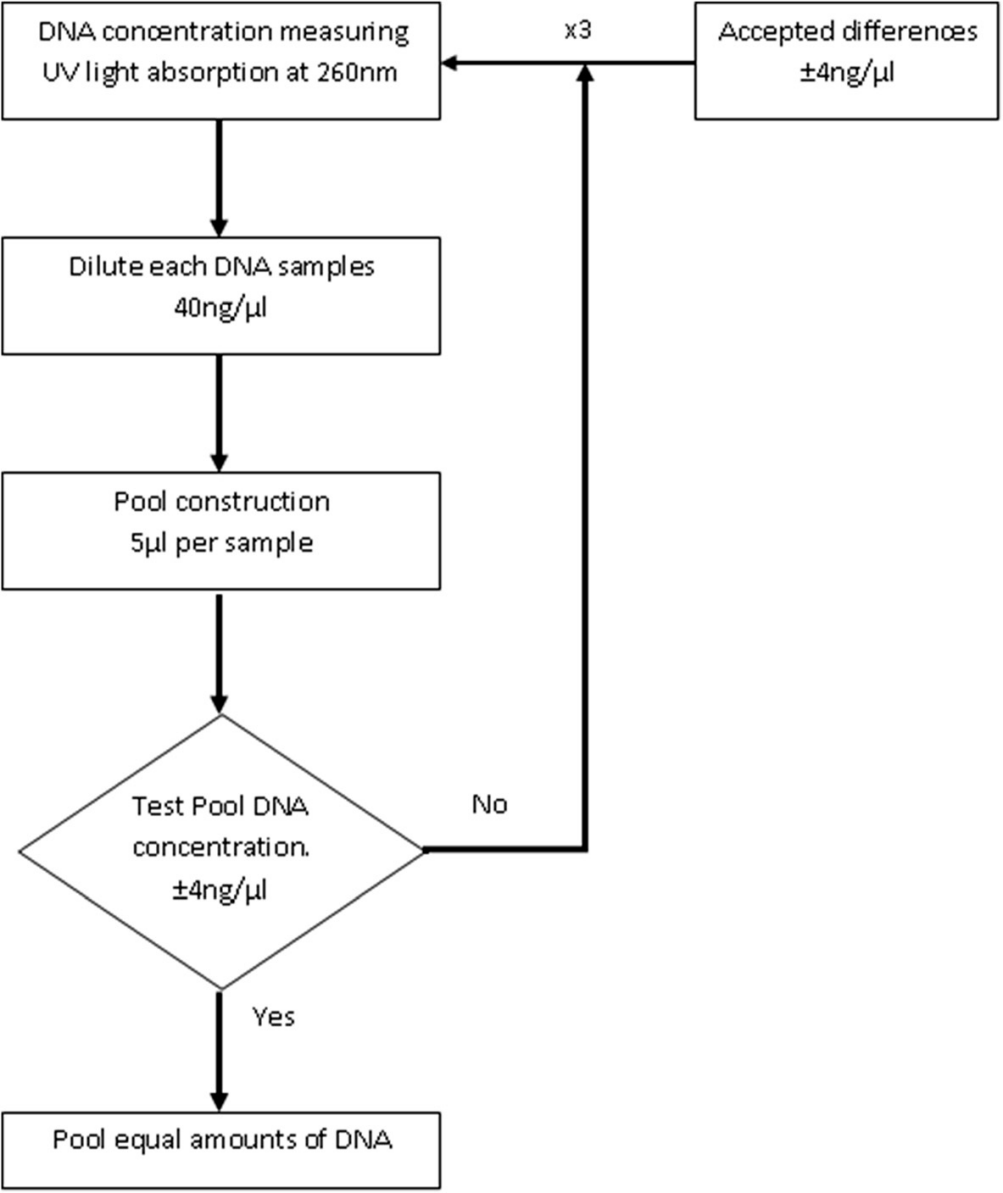
Protocol for performing DNA Pools. First, DNA concentrations of individual samples were measured three times by ultraviolet (UV) light absorption (Nanodrop spectrophotometer). When readings differ less than 4 ng/μl, samples were diluted to 40 ng/μl. Next, the pools were prepared using 5ul of DNA from each of the 5 samples that would form part of the pool. The final concentration of every pool should be 25 μl at 40 ng/μl, with DNA concentration checked by UV light absorption (Nanodrop spectrophotometer) again. Finally, if readings were 40 ng/μl ±4 ng/μl, pools were analysed with the EWAS arrays. If readings differed, the pools were created again from the first step

DNA concentrations for each subject were determined individually, by measuring ultraviolet (UV) light absorption at 260 nm, with NanoDrop 2000 UV–vis Spectrophotometer (Thermo Scientific, Redwood City, CA, USA). Adapting the instructions of previous DNA pooling protocols [14, 15], each sample was diluted to 40 ng/ul, and their DNA concentration was measured again to verify that all samples provide the same amount of DNA to the pools. Samples with DNA concentration variations higher than 40 ng/μl ±4 were discarded and diluted again. Individual DNA samples were then added to their respective pool (4 μl at 40 ng/μl of each sample). Once each pool was generated, the DNA concentrations were re-quantified twice with NanoDrop to assure that the final concentration of the pool was as expected (40 ng/μl). If any discrepancy was found (> ± 4 ng/μl), the pool was generated again repeating all steps from sample DNA measures. Only when the final pool concentration was 40 ng/μl ±4 ng/μl and the total volume was 20 μl as expected was the EWAS analysis started. A graphical description of the procedure can be found in Fig. 5.

### Epigenome wide association analysis

Genome-wide DNA methylation was assessed using the Infinium HumanMethylation450 BeadChip (Illumina Inc., San Diego, Ca). This chip-based study quantitatively measures more than 450,000 CpG sites at single nucleotide resolution with a 99 % coverage of RefSeq Genes.

A Quality Control (QC) of all samples was performed as a first step to check DNA integrity using Invitrogen E-Gel 1 % Agarose Gels. The DNA samples showed no fragmentation or poor quality.

Genomic DNA from the 20 samples and the 4 pools was bisulphite converted using the Zymo EZ DNA Methylation^TM^ Kit (Zymo Research, Orange, Ca) following the manufacturer’s instructions, but with alternative incubation conditions suggested for the Illumina Infinium Methylation Assay. All samples were processed in a single working batch using the Illumina Infinium MSA4 protocol, which includes amplification, fragmentation, hybridization and BeadChip scanning.

For QC, the fluorescence data generated for each CpG locus was analysed with the Illumina GenomeStudio software package. Samples and CpG sites with fluorescence detection p-values > 0.05 were removed [22]. This p-vaule is the detection p-value that represents the confidence that a given methylation level on a CpG site can be considered to have been detected.

### Quality control and normalization

All pre-processing, correction and normalization steps were implemented using the R computing environment (versions 2.15.1 and 3.0.1) with Bioconductor packages. Plots were produced using R functions. The pipeline was a sequence of R scripts adapted from the methylumi [23] (version 2.6.1), lumi [24] (version 2.12.0), watermelon [25] (version 1.0.3) and minfi [22] (version 1.6.0) packages. The instructions that were used are shown in Table 2.

**Table 2:** R packages and instructions. Specific instructions used from each R package

R package	Instruction	Description
methylumi	methylumiR	Load Illumina methylation data into a MethyLumiSet object.
minfi	densityPlot	Density plots of methylation Beta values.
watRmelon	pfilter	Filter data sets based on bead count and detection p-values
minfi	mdsPlot	Multi-dimensional scaling (MDS) plots showing a 2-d projection of distances between samples.
watRmelon	dasen	Calculate normalized betas from Illumina 450 K methylation arrays.
lumi	estimateBetas	Estimate methylation Beta-value matrix from eSet-class object (include methylated and unmethylated probe intensities)

Prior to the identification of differentially methylated CpG sites, data was pre-processed using a non-specific filter step. This step consists of removing CpG sites with detection p-value ≥ 0.05 in more than 1 % of the samples. Samples with detection p-value ≥ 0.05 in more than 1 % of the CpG sites, and CpG sites with beadCount < 3 in 5 % of samples [16]. CpG sites containing documented single nucleotide polymorphisms (SNPs) were also removed [26]. Multidimensional scaling (MDS) plots were used to evaluate gender outliers based on chromosome X data, where males and females were separated into two distinct clusters. An MDS plot was also used to check for unknown population structures, inside the sample. Then, CpG sites on the X and Y chromosomes were removed [8]. Finally, a subset quantile normalization was performed using a background adjustment between-array normalization and a dye bias correction, following previous recommendations [27, 28].

### Statistical analysis

All statistical analysis was also performed using R (version 3.0.1). The accuracy of DNA methylation level estimations from pooled DNA was assessed with a Spearman’s correlation, for non-parametric samples, between the β-values of each pool and the averaged β-values of the individual samples included in each pool [19].

We also performed a Spearman’s correlation between the β-values of the 101 most differentially methylated CpGs (DMCs) found in individual samples (Group A vs. Group B) and the β-values of the same CpG sites in pools. Differentially methylated CpG sites were determined by the Mann–Whitney U-test for non-parametric samples using the β-values, *p*-val < 10^−06^ adapted from Rakyan VK el al. [4]. The DMCs analysis was performed comparing group A samples (*n* = 10) against group B samples (*n* = 10).

Minimum sample size needed for pool analysis in EWAS was calculated using the *pwr* package [29] with implemented power analysis as outlined by J. Cohen, 1988.

### Ethical considerations

Ethical approval has been obtained from the ethical committee of the Vall d’Hebron Hospital (PR(AG) 03/2007). All patients were provided with oral and written information about the project, and each participant signed the informed consent for the study.
